# Circulating Extracellular Vesicles microRNAs Are Altered in Women Undergoing Preterm Birth

**DOI:** 10.3390/ijms24065527

**Published:** 2023-03-14

**Authors:** Bruna Ribeiro Andrade Ramos, Júlia Abbade Tronco, Márcio Carvalho, Tainara Francini Felix, Patrícia Pintor Reis, Juliano Coelho Silveira, Márcia Guimarães Silva

**Affiliations:** 1 Department of Pathology, Botucatu Medical School, São Paulo State University (UNESP), Botucatu 17213-700, SP, Brazil; 2 Faculty of Medicine—Jaú Campus, University of Western São Paulo (UNOESTE), Jaú 17213-700, SP, Brazil; 3 Faculty of Veterinary Medicine and Animal Science, São Paulo State University (UNESP), Botucatu 17213-700, SP, Brazil; 4 Experimental Research Unity (UNIPEX), Botucatu Medical School, São Paulo State University (UNESP), Botucatu 17213-700, SP, Brazil; 5 Department of Surgery and Orthopedics, Botucatu Medical School, São Paulo State University (UNESP), Botucatu 17213-700, SP, Brazil; 6 Department of Veterinary Medicine, Faculty of Animal Science and Food Engineering, São Paulo University (USP), Pirassununga 13635-900, SP, Brazil

**Keywords:** preterm birth, preterm labor, preterm premature rupture of membranes, microRNAs, small extracellular vesicles, inflammation

## Abstract

Preterm labor (PTL) and preterm premature rupture of membranes (PPROM) lead to high perinatal morbidity/mortality rates worldwide. Small extracellular vesicles (sEV) act in cell communication and contain microRNAs that may contribute to the pathogenesis of these complications. We aimed to compare the expression, in sEV from peripheral blood, of miRNAs between term and preterm pregnancies. This cross-sectional study included women who underwent PTL, PPROM, and term pregnancies, examined at the Botucatu Medical School Hospital, SP, Brazil. sEV were isolated from plasma. Western blot used to detect exosomal protein CD63 and nanoparticle tracking analysis were performed. The expression of 800 miRNAs was assessed by the nCounter Humanv3 miRNA Assay (NanoString). The miRNA expression and relative risk were determined. Samples from 31 women—15 preterm and 16 term—were included. miR-612 expression was increased in the preterm groups. miR-612 has been shown to increase apoptosis in tumor cells and to regulate the nuclear factor κB inflammatory pathway, processes involved in PTL/PPROM pathogenesis. miR-1253, miR-1283, miR378e, and miR-579-3p, all associated with cellular senescence, were downregulated in PPROM compared with term pregnancies. We conclude that miRNAs from circulating sEV are differentially expressed between term and preterm pregnancies and modulate genes in pathways that are relevant to PTL/PPROM pathogenesis.

## 1. Introduction

Preterm labor (PTL)—defined as labor before gestational week 37—is a worldwide health concern that affects around 10% of all pregnancies [1,2,3]. This condition along with preterm premature rupture of membranes (PPROM)—characterized by the rupture of fetal membranes before gestational week 37—are the main causes of spontaneous preterm birth (PTB). Despite the intensive efforts of researchers to fully elucidate the pathophysiology of PTL and PPROM and to avoid their occurrence, such disorders continue to be clinically challenging and represent a significant burden to preterm infants, their families, and health systems around the world. Indeed, preterm infants have an increased risk of pulmonary dysplasia, cognitive disorders, and infections, among other morbidities, when compared with term newborns [4,5]. Moreover, prematurity can cause lifetime complications, such as increased cardiovascular risk and neurodevelopment impairment [6,7]. The World Health Organization (WHO) estimates that 28% of early neonatal deaths—excluding malformation—and 17% of deaths among children under 5 years old result from PTB [8].

Several risk factors, such as intrauterine infections, bacterial vaginosis, genetic predisposition, and behavioral habits, have already been associated with PTL and PPROM [9,10,11]. These factors are influenced by epigenetic factors such as small non-coding RNAs. MicroRNAs (miRNA) post-transcriptionally regulate 50–60% of the human genome [12,13] and have already been described to play an important role in different stages of pregnancy, from implantation to parturition, both in physiological and pathological conditions [14,15,16,17,18,19]. For example, the reduced placental expression of 15 miRNAs from the C19MC placental cluster is associated with gestational hypertension, intrauterine growth restriction, and preeclampsia [20]. Similarly, the increased expression of miR-145, miR-143 and miR-199 in cervical cells is associated with PTL [21]; this study showed that high levels of miR-145 reduce the expression of adhesion proteins, increasing the permeability of the cervical epithelial barrier and contributing to cervical effacement. Another study identified significant differences in miRNA expression in whole blood samples from women who underwent PTL, compared with women who underwent term birth. Most of these miRNAs were predicted to target genes previously described to be associated with PTL pathways, such as interleukin production and activation of the immune system [22].

In this context, miRNAs may help us to elucidate the undetermined molecular pathways linked to PTB. Their appeal relies on their abundance in tissues and body fluids, stability, and ease of access, especially when confined to circulating small extracellular vesicles (sEV) such as exosomes [23,24]. Exosomes are double-membrane nanometric structures that act in cell–cell communication, carrying proteins and nucleic acids from one cell type to another in a specific manner [25]. sEV are secreted by trophoblasts and placental and fetal cells [26] and have recently been shown to play important roles in maternal–fetal communication [27]. Indeed, sEV play a critical role in several gestational diseases, from gestational diabetes to PTB, as they modulate maternal immunologic responses [28,29]. In an experimental model, sEV have been demonstrated to migrate from maternal circulation to the fetus and to induce PTL, thus working as a paracrine mediator of labor [27].

Considering the high stability and relevance of the sEV cargo, studying miRNAs is critical to fill the knowledge gap concerning the pathophysiology of PTL and PPROM. To the best of our knowledge, there are no studies that have addressed sEV-derived miRNAs in a Brazilian population. Thus, we aimed to evaluate the expression of miRNAs isolated from circulating sEV in preterm pregnancies in a Brazilian population. We found that miRNAs from circulating sEV are differentially expressed between term and preterm pregnancies and modulate genes in pathways relevant to PTL/PPROM pathogenesis.

## 2. Results

### 2.1. Patients

The sociodemographic and clinical characteristics of women included in the study are displayed in Table 1. The overall mean age was 26.4 ± 5.5 years. There were no significant differences regarding age, body mass index, type of labor, smoking status, or history of previous abortion among the groups. The mean gestational age at birth was 35 weeks and 2 days ± 2 weeks and 6 days for the PTL group; 34 weeks and 1 day ± 1 week and 6 days for the PPROM group; 39 weeks and 6 days ± 1 week and 2 days for the term in labor (TL) group; and 38 weeks and 6 days ± 3 days for the term out of labor (T) group. The time between the onset of PTL or PPROM and labor was 19.5 ± 18.6 and 12.9 ± 15.3 days, respectively. A personal or family history of prematurity and self-reported white ethnicity was associated with the PTL group (*p* = 0.02 and *p* = 0.03, respectively). Being single was associated with the PPROM group (*p* = 0.001). Indications for cesarean section included repeated previous cesarean sections, breech presentation, and labor dystocia. Clinical data on the newborns are presented in Table 2. As expected, birth weight was higher in the TL and T groups compared with the PTL and PPROM groups (*p* = 0.004).

### 2.2. sEV Characterization

**Figure 1:**
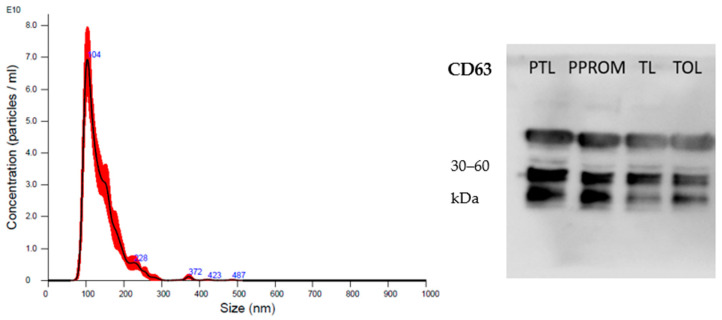
sEV characterization by nanotracking particle analysis—size distribution and concentration of small extracellular vesicles (sEV) isolated from plasma—and Western blotting for the exosome marker CD63.

Based on the BCA assay, the mean protein concentration in sEV was 912.5 ± 152.0 µg/mL. Successful sEV isolation was confirmed by nanoparticle tracking analysis (NTA) and Western blotting analysis (Figure 1). The mean particle mode size of the isolated vesicles was 98.0 ± 16.9 nm, which is compatible with the size of exosomes/sEV, and the mean concentration of particles was 1.71 × 10^12^ particles/mL. We also evaluated whether women undergoing PTL or PPROM produce higher circulating levels of sEV than those who underwent term labor. NTA analysis revealed that women undergoing PTL or PPROM do not have higher circulating levels of sEV. Table 3 shows the protein concentration and NTA data.

We identified the exosome marker CD63 in the isolated sEV samples and, as expected, the negative control cytochrome *c* was absent. Figure 1 shows the CD63 bands in the Western blot analysis (26 kDa, with glycosylated variations of 30–60 kDa).

### 2.3. miRNA Expression

Among the 800 miRNAs we investigated, 12 presented high counts (Table 4). miR-6721 counts were stable among all the samples; therefore, we used this miRNA as an endogenous control for data normalization.

miR-612 expression was higher in the PTL and PPROM groups compared with the TL and T groups. miR-1253, miR-1283, miR378e, and miR-579-3p showed decreased expression in the PPROM group compared with the T group. When we compared only the prematurity groups (PTL vs. PPROM), most miRNAs were differentially expressed (Table 4). When we compared only the term groups (TL vs. T), miR-302-3p and miR-612 were higher during labor, while miR-451a and miR-520f were higher in not in labor (Table 4).

### 2.4. Enriched Pathways

**Figure 2:**
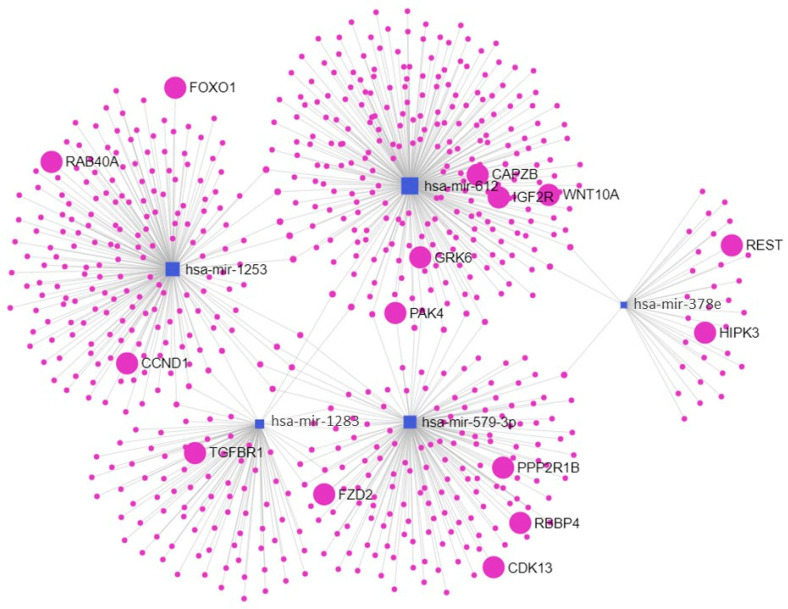
miRNA-target gene interaction networks. Squares represent the differently expressed miRNAs (miR-612, miR-1253, miR-1283, miR-378e, and miR-579-3p). Circles represent target genes and highlighted circles (larger circles) represent genes from major pathways involved in prematurity. This figure was generated using the miRNet 2.0 tool (https://www.mirnet.ca/ accessed on 13 February 2023).

Computational analysis revealed that miR-612 target genes are related to endocytic pathways (Table 5) and that miR-1253, miR-1283, miR378e, and miR-579-3p act in cellular senescence pathways (Table 6) (Figure 2).

Furthermore, gene ontology analysis of the target genes revealed their role as regulators of several biological processes, such as endocytosis (GO:0045806), phagocytosis (GO:0050766), cellular senescence (GO:2000773), cell–cell adhesion (GO:0022407), and apoptotic process (GO:0042981).

Detailed results can be found at https://maayanlab.cloud/Enrichr/enrich?dataset=4586cd54f00f6cdba2ab4bbf602325ca and https://maayanlab.cloud/Enrichr/enrich?dataset=a4c309d2b4db51232173445e28b646b3 accessed on 18 August 2022 (Kyoto Encyclopedia of Genes and Genomes (KEGG) 2021 Human database). The complete gene ontology of the target genes is provided in the Supplementary Table S1.

## 3. Discussion

miRNAs have fundamental roles in distinct biological processes. However, it was not until recently that these molecules began to be investigated more thoroughly in the context of maternal–fetal disorders. In the present study, we have reported the altered expression of five miRNAs from peripheral blood sEV from women undergoing PTL and PPROM.

We detected increased miR-612 expression in the PTL and PPROM samples. Although there have been no reports of this miRNA in the gestational context, it is possible to draw a parallel with the existing literature. miR-612 presents anti-tumorigenic effects in cancer [30,31] by inducing increased apoptosis of tumor cells [32]. p53 is a pivotal protein for apoptotic pathways and the 3′ untranslated (UTR) region of *TP53* messenger RNA (mRNA) is a miR-612 target [33]. Additionally, a recent study demonstrated that this miRNA regulates the nuclear factor κB (NF-κB) inflammatory pathway [32]. Both apoptosis and NF-kB activation are linked to the pathogenesis of PTL and PPROM. Computational analysis revealed that target genes for miR-612 are involved in the endocytosis and phagocytosis pathways. Endocytic and phagocytic pathways are pivotal for the elimination of extracellular pathogens and have been shown to be present among amniotic fluid neutrophils [34]. We hypothesize that the disruption of these pathways renders gestational tissues more vulnerable to subclinical infection and inflammation, possibly prematurely triggering labor pathways [35].

We also detected the decreased expression of miR-1253, miR-1283, miR-378e, and miR-579-3p in women undergoing PPROM. Our report is the first to show the altered expression of these miRNAs in blood samples from women with PPROM. Studies conducted with first-trimester placenta samples have demonstrated that miR-1283 is involved in trophoblast proliferation [36] and that the cell cycle regulator CCD1 is a target of miR-1283 [37]. Cell proliferation is an important feature for the maintenance of chorioamniotic membrane integrity. In general, there are limited data regarding miR-1253, miR-378e, and miR-579-3p, and there are no data concerning their role in pregnancy complications. The pathway analysis results are in accordance with the role described above, as we observed that this set of miRNAs act in pathways linked to cellular senescence [38]. The increased senescence of fetal membranes is the underlying mechanism implicated in PPROM pathogenesis [39,40].

Some miRNAs were associated with the occurrence of labor itself rather than the time of delivery, such as miR-302-3p, miR-451a, and miR-520f. The levels of these miRNAs did not differ between the preterm and term groups; however, their levels were significantly different between the presence or absence of labor. Animal model studies have suggested an important role for high miR-451a levels during the implantation period [41], an observation reinforced by clinical studies [42]. However, there is a lack of data concerning the third gestational trimester. In the present study, we have reported the elevated expression of miR-451a and miR-520f in women not in labor in both pathological (PPROM) and physiological (T) conditions. Elevated levels of these miRNAs may indicate failure in triggering labor pathways. Considering the great number of predicted targets for each miRNA detected in the present study (there are over 1360 predicted targets for miR-1283—for example, see miRdb.com), future functional studies are needed to reiterate the suggested mechanisms underlying the associations herein observed.

Concerning the quantity of sEV and corroborating the literature [43], the number of particles detected was similar among the studied groups regardless of the gestational age, and presence or absence of labor. This demonstrates only their cargo, rather than their production, was associated with the different gestational outcomes. Regarding the evaluated social and clinical parameters, in accordance with the reported genetic predisposition of prematurity [44], a personal or family history of this condition was associated with the (re)occurrence of PTL. Two other factors reported to be associated with preterm outcomes were self-reported white ethnicity and single status. We have previously shown that our population (i.e., Brazilian) behaves differently from those reported in other countries regarding the relationship between ethnicity and PTB predisposition, probably due to the distinct environments and genetic background [44]. Considering marital status, it has been hypothesized that being single increases maternal stress, which is a known risk factor for preterm outcomes [45]. Nevertheless, it is important to highlight that maternal stress is a multifactorial situation that we have not addressed in our study.

Our study is not the first to attempt to identify a miRNAome signature for PTB in peripheral blood samples [23,46,47]. In recent work, Menon et al. [46] reported a differentially expressed miRNA profile in circulating exosomes throughout pregnancy. Bioinformatics analysis of the identified miRNAs has pointed to the regulation of the pathways involved with transforming growth factor beta, p53, and glucocorticoid signaling. In a retrospective case–control study, Winger et al. [23] identified the risk of PTB in first-trimester blood samples in an African American population, based on the quantification of 45 selected miRNAs.

A limitation of the present study is that we did not tag sEV; thus, we cannot infer the origin of the studied vesicles—whether they were derived from the placenta or originated from other sites. Nevertheless, this does not reduce the relevance of our data, because our objective was to detect miRNAs from sEV that were differently expressed in women undergoing PTL and PPROM, regardless of the origin of the extracellular vesicles. Another limitation is the lack of functional data; thus, our next step is to evaluate the miRNAs discussed here in vitro to determine their effects on mRNA and protein expression. Functional studies on in vitro two-dimensional cultures and organ-on-a-chip models are needed to effectively determine whether there is a causality link between these miRNAs and the occurrence of PTL and PPROM or whether their differential expression is rather a consequence of the labor pathways that have been set in motion. Moreover, while the thorough inclusion and exclusion criteria were intended to yield a homogenous sample, we cannot completely overrule the possibility of confounding factors in our sample.

A strength of our study Is that, to our knowledge, it is the first to evaluate the sEV miRNA signature for PTL and PPROM in a Brazilian population, a relevant topic considering the burden of prematurity in our population and the heterogeneity of these conditions among distinct populations.

In addition, we used a highly sensitive and specific platform, the Nanostring nCounter assay, for a comprehensive panel of 800 miRNAs that have been fully annotated in miRBase (https://www.mirbase.org/ accessed on 18 August 2022). The analysis was performed by signal counting, directly quantifying the sequences of interest. This strategy avoids the bias of amplification-based methods, generating reproducible results. Various studies have demonstrated its sensitivity, specificity and reproducibility compared to other methods [48,49].

In conclusion, miRNAs from circulating sEV are differently expressed between term and preterm pregnancies and modulate genes in the pathways relevant to PTL/PPROM pathogenesis. Future in vitro studies will allow us to elucidate the exact role of inflammatory and senescence-related miRNAs, among other miRNAs, in the pathophysiology of PTL and PPROM.

## 4. Materials and Methods

### 4.1. Patients

This cross-sectional study included 31 pregnant women, recruited by convenience sampling, examined at the Clinical Hospital of Botucatu Medical School and the city’s Basic Health Units, SP, Brazil, from January 2017 to August 2019. Gestational age was calculated based on the date of their last period and/or early ultrasound. Samples were allocated into the PTL, PPROM, TL, and T groups. The groups were defined according to the guidelines of the Brazilian Ministry of Health. PTL was defined as the presence of regular uterine contractions every ≤10 min and cervical effacement ≥50% was confirmed by two observers and/or cervical dilatation of at least 2 cm at <37 weeks of gestation. TL was defined by the same parameters described above at ≥37 weeks of gestation. PPROM was defined by the spontaneous rupture of fetal membranes before labor at <37 weeks of gestation. Finally, T was defined by the absence of clinical signs of labor at ≥37 weeks of gestation [50]. The exclusion criteria included multiple pregnancies, gestational pathologies (preeclampsia, gestational hypertension, gestational diabetes, cervical insufficiency, placental abruption, clinical chorioamnionitis and fetal growth restriction), genetic abnormalities (trisomies), and systemic diseases or infections (e.g., coagulation disorders and HIV). Additionally, we had no IVF patients in our setting.

The research was approved by the Ethics Research Committee of Botucatu Medical School, UNESP (CAAE 61138116.8.0000.5411), and all the patients signed a written informed consent form. We confirm that this research was performed following the relevant guidelines and regulations.

### 4.2. Sample Collection

Peripheral blood samples were collected using EDTA sterile tubes upon the patient’s admission to the Obstetrics Services (PTL (*n* = 8), PPROM (*n* = 7), TL (*n* = 9), and T (*n* = 7)). A questionnaire was used to obtain sociodemographic and clinical data. To maintain the homogeneity of the samples, the patients should have fasted for at least 1 h prior to blood collection and all the samples were collected between 9:30 and 10:30 am because the circadian cycle may influence exosome release [51]. The first 2 mL of blood was collected in a separate tube that was discarded, following the recommendation from the International Society for Extracellular Vesicles (ISEV), and the samples were visually inspected for hemolysis [51]. Plasma was obtained by centrifugation at 1800 g for 10 min at room temperature and stored at −80 °C for up to 2 weeks until sEV isolation.

### 4.3. Definition and Isolation of sEV

Following the International Society for Extracellular Vesicles (ISEV) endorsement, we adopted the terminology small extracellular vesicles (sEV) for the particles naturally released from the cell that are delimited by a lipid bilayer, cannot replicate, and are <200 nm [51].

sEV were isolated from 1 mL of plasma using the Total Exosome Isolation Reagent (from plasma) (Invitrogen—MA, USA), following the proteinase K protocol, which is recommended for downstream applications other than protein analysis. For two samples of each group, an additional aliquot of 250 µL was used to isolate exosomes without adding proteinase K to evaluate the exosomal surface proteins by Western blot (Figure 1).

### 4.4. Characterization of sEV

The indirect quantification of proteins was performed using the Pierce BCA Protein Kit (Thermo Scientific—MA, USA). NTA was performed using Nanosight NS300 with the following parameters: 38.5 °C, capture of 30 s, and 5 reads.

Western blot was performed to detect the exosome marker CD63; the negative control was cytochrome c (Santa Cruz Biotechnology, Inc.—Texas, USA). This step was performed jointly with another study from our group that has already been published [52]. Briefly, 4× Laemmli buffer and mercaptoethanol (1:10) were used for protein extraction (5 min at 95 °C). The protein concentration was quantified with the Pierce BCA Assay kit and 5 µg of protein was added to each lane of a polyacrylamide gel (separation gel 12%, stacking gel 4%). The protein was separated at 100 V for 140 min. The separated protein was transferred to a nitrocellulose membrane at 80 V for 120 min. The nitrocellulose membrane was incubated with a solution that contained the primary antibody in 1× Tris-buffered saline with Tween 20 (TBS-T) and 1% bovine serum albumin (BSA) overnight at 4 °C. Then, the membrane was incubated in a solution that contained the secondary antibody in 1× TBS-T 1× and 5% BSA. The data were analyzed with ImageQuant LAS 4000 software version 1.2.

### 4.5. Total RNA Extraction and Purification

The RNA/DNA/Protein Purification Plus Kit (Norgen Biotek—Thorold, Canada) was used to extract the total RNA from the sEV samples. After the lysis step of the recommended protocol, 5 µL of spike-in Ath-miR-159a at 200 pM was added. The total RNA was eluted in 50 µL, quantified using a Nanodrop and Qubit High Sensitivity RNA kit (Life Technologies—MA, USA), and stored at −80 °C. The samples were purified and concentrated using Amicon Ultra 0.5 mL 3 KDa ultrafiltration columns (Millipore—MA, USA) for 90 min at 14,000 g, and then combined into pools.

### 4.6. NanoString nCounter Profiling Analysis

miRNA expression was evaluated using the nCounter Human V3 miRNA Assay (NanoString Technologies—Seattle, WA, USA), a highly specific and sensitive platform that analyzes 800 miRNAs. Nanostring is a color-coded probe-based assay based on nucleic acid hybridization specific to capture target sequences without biases inherent to amplification-based assays. This analysis followed previously reported studies [53,54]. Briefly, upon adapter ligation and hybridization of the CodeSets, the samples were placed in the Prep Station and scanned in the Digital Analyzer using 555 fields of view (FOV). The data were analyzed using the nSolver Analysis software version 2.0.134.

### 4.7. Pathway Enrichment and Network Analyses

Differentially expressed miRNAs were subjected to target prediction analysis using the microRNA Data Integration Portal (miRDIP) (http://ophid.utoronto.ca/mirDIP/ version 5.0.2.3, accessed on 18 August 2022). The microRNA–gene matrix was applied, and the criteria for target identification were set at a “high” score for predicted interactions [55]. After identifying the predicted miRNA target genes, pathway enrichment analysis was performed with the Enrichr tool (http://amp.pharm.mssm.edu/Enrichr, accessed on 18 August 2022) [56,57,58] and the KEGG 2021 Human database was used to determine the enriched pathways and gene ontology/biological roles of the miRNA target genes. miRNet tool (https://www.mirnet.ca/ version 2.0, accessed on 18 August 2022) [59,60,61] was used to visualize miRNA-target mRNA interaction networks.

### 4.8. Statistical Analysis

The sample size (*n* = 7 for each group) was calculated a priori considering an α of 5% and power (1-β) of 80% for detecting a difference of two standard deviations between the groups.

Continuous variables regarding the sociodemographic and clinical data—age, gestational age, body mass index (BMI), newborn weight, and Apgar—passed the D’Agostino–Pearson normality test and were compared using analysis of variance (ANOVA), followed by Tukey’s multiple comparison test. Categorical variables were compared by the chi-square test or Fisher’s exact test (cell size < 5). The analysis was performed with GraphPad Prism 5.0.

miRNA data were analyzed in two stages, both using NanoString’s nSolver Analysis and SAS 3.0 software. First, in nSolver, the option “threshold background” was selected using negative controls and the counts were normalized using miR-6721 as an endogenous control, as the expression of this miRNA was even more stable than of the exogenous miRNAs added to the samples. Then, the comparison of miRNA expression among the groups and the relative risk was performed using a generalized linear model with a Poisson or a negative binomial distribution, according to the overdispersion, followed the Wald multiple comparison test. A *p*-value of 0.05 was considered as statistically significant.

## Figures and Tables

**Table 1:** Sociodemographic and clinical characteristics of women included in the study.

Variables	PTL (*n* = 8)	PPROM (*n* = 7)	TL (*n* = 7)	T (*n* = 9)	*p*
Age (years) *	25.6 ± 4.2	26.0 ± 7.4	23.8 ± 4.6	29.3 ± 4.9	NS
GA at delivery (days) *	247.0 ± 10.2 ^a^	239.6 ± 13.3 ^a^	279.9 ± 9.8 ^b^	272.1 ± 3.8 ^b^	<0.0001
BMI (kg/h^2^) *	26.6 ± 3.8	29.6 ± 7.3	27.3 ± 3.1	28.6 ± 5.1	NS
Delivery (%)					
Vaginal	67 (4/6)	57 (4/7)	43 (3/7)	-	NS
Caesarean	33 (2/6)	43 (3/7)	57 (4/7)	100 (9/9)	
Marital status (%)					
Single	13 (1/8)	71 (5/7)	-	-	0.001
Civil union	87 (7/8) ^a^	29 (2/7)^b^	100 (7/7) ^a^	100 (9/9) ^a^	
Self-reported ethnicity (%)					
White	75 (6/8) ^a^	57 (4/7) ^b^	14 (1/7) ^b^	67 (6/9) ^b^	0.03
Non-white	25 (2/8)	43 (3/7)	86 (6/7)	33 (3/9)	
Parturity (%)					
First pregnancy	25% (2/8)	57 (4/7)	57 (4/7)	44 (4/9)	NS
Multiple pregnancies	75% (6/8)	43 (3/7)	43 (3/7)	56 (5/9)	
Smoking (%)					
Smoking	-	14 (1/7)	-	11 (1/9)	NS
Not smoking	100 (8/8)	86 (6/7)	100 (7/7)	89 (8/9)	
Years of study (%)					
<8 years	-	14 (1/7)	-	11 (1/9)	NS
≥8 years	100 (8/8)	86 (6/7)	100 (7/7)	89 (8/9)	
Previous history of PTL/PPROM (%)					
Presence	38 (3/8)	-	-	-	
Absence	62 (5/8) ^b^	100 (7/7)^a^	100 (7/7) ^a^	100 (9/9) ^a^	0.02
Prior abortion (%)					
Presence	38 (3/8)	-	-	22 (2/9)	NS
Absence	62 (5/8)	100 (7/7)	100 (7/7)	88 (7/9)	

PTL: preterm labor; PPROM: preterm premature rupture of membranes; TL: term in labor; T: term out of labor GA: gestational age; BMI: body mass index; NS: non-significant (*p* > 0.05). The letters “a” and “b” represent statistical differences. * The data are presented as mean ± standard deviation and were compared by analysis of variance, followed by Tukey’s multiple comparison test. Qualitative variables were analyzed by the chi-square test or Fisher’s exact test (cell size < 5).

**Table 2:** Clinical data on the newborns included in this study.

Variables	PTL (*n* = 8)	PPROM (*n* = 7)	TL (*n* = 7)	T (*n* = 9)	*p*
Weight (g) *	2409 ± 677.0 ^a^	2208 ± 401.6 ^a^	3256 ± 459.1 ^b^	3358 ± 470.2 ^b^	0.004
Apgar 10 *	9.3 ± 0.8	9.0 ± 0.9	9.7 ± 0.5	9.8 ± 0.4	NS
Sex (%)					
Female	38 (3/8)	14 (1/7)	43 (3/7)	56 (5/9)	NS
Male	62 (5/8)	86 (6/7)	57 (4/7)	44 (4/9)	

PTL: preterm labor; PPROM: preterm premature rupture of membranes; TL: term in labor; T: term out of labor; GA: gestational age; BMI: body mass index; NS: non-significant (*p* > 0.05). The letters “a” and “b” represent statistical differences. * The data are presented as mean ± standard deviation and were compared by analysis of variance, followed by Tukey’s multiple comparison test.

**Table 3:** Comparison of protein quantification and nanoparticle tracking analysis data.

Variables	Protein (µg/mL)	Particles/mL *	Mode	*p*
PTL	2964.1 ± 1595	9.85 × 10^11^	105.2 ± 13.5	NS
PPROM	2528.0 ± 1092	5.81 × 10^11^	107.6 ± 11.5	
TL	2576.5 ± 1940	5.60 × 10^11^	103.8 ± 12.0	
T	2338.9 ± 926	5.05 × 10^11^	101.8 ± 7.9	

PTL: preterm labor; PPROM: preterm premature rupture of membranes; TL: term in labor; T: term out of labor; NS: non-significant (*p* > 0.05). The mean protein concentration (µg/mL) was determined with the Pierce BCA protein assay. * The data are presented by mean ± standard deviation and compared by analysis of variance. The *p*-value represents the comparison of particles/mL among the groups.

**Table 4:** Mean count of microRNAs (miRNAs; hsa-miR) and comparison among groups.

miRNAs	PTL (*n* = 8)	PPROM (*n* = 7)	TL (*n* = 7)	T (*n* = 9)	PTL vs. TL	PPROM vs. T	PTL vs. PPROM	TL vs. T
let-7i-5p	831.2 ± 84.3	527.7 ± 121.1	814.7 ± 86.8	742.1 ± 154.9	NS	NS	RR = 1.54 (1.16–2.05)	NS
miR-1253	224.0 ± 28.2	153.9 ± 8.4	214.6 ± 32.0	211.3 ± 21.9	NS	RR = 0.73 (0.65–0.82)	RR = 1.47 (1.30–1.65)	NS
miR-1283	134.1 ± 20.4	83.4 ± 16.6	122.0 ± 16.8	125.4 ± 11.4	NS	RR = 0.68 (0.58–0.78)	RR = 1.60 (1.37–1.87)	NS
miR-302-3p	259.5 ± 40.8	201.0 ± 28.0	236.0 ± 36.9	196.7 ± 16.4	NS	NS	RR = 1.29 (1.02–1.64)	RR = 1.21 (1.08–1.35)
miR-3144-3p	103.6 ± 16.7	80.0 ± 7.0	96.8 ± 12.0	85.8 ± 9.4	NS	NS	RR = 1.31 (1.11–1.55)	NS
miR-362-3p	162.1 ± 24.2	140.2 ± 16.6	154.0 ± 9.5	145.2 ± 15.2	NS	NS	RR = 1.16 (1.02–1.32)	NS
miR-378e	301.1 ± 31.9	200.9 ± 19.6	284.7 ± 34.1	279.4 ± 38.0	NS	RR = 0.71 (0.65–0.79)	RR = 1.50 (1.35–1.66)	NS
miR-451a	35.6 ± 7.8	188.2 ± 53.6	40.0 ± 12.1	122.6 ± 72.6	NS	NS	RR = 0.19 (0.12–0.29)	RR = 0.30 (0.15–0.59)
miR-520f	28.4 ± 3.7	83.8 ± 62.8	32.0 ± 2.6	96.9 ± 87.9	NS	NS	RR = 0.28 (0.13–0.60)	RR = 0.26 (0.12–0.58)
miR-579-3p	2219.4 ± 438.1	1188.3 ± 206.8	2002.9 ± 451.3	2266.4 ± 479.0	NS	RR = 0.52 (0.38–0.71)	RR = 1.88 (1.38–2.54)	NS
miR-612	187.7 ± 22.4	168.7 ± 18.6	155.7 ± 24.4	135.3 ± 16.7	RR = 1.20 (1.06–1.36)	RR = 1.25 (1.09–1.42)	NS	RR = 1.16 (1.01–1.32)

PTL: preterm labor; PPROM: preterm premature rupture of membranes; TL: term in labor; T: term out of labor; NS: non-significant (*p* > 0.05). miRNA counts and relative risk were analyzed using a generalized linear model (Poisson distribution or negative binomial), followed by the Wald multiple comparison test.

**Table 5:** Main pathways regulated by miR-612.

Pathways	*p*	Genes
Endocytosis	1.48 × 10^12^	*EHD2; TSG101; SMURF1; CAV1; CAPZ; GRK6; PSD3; CHMP7; RAB; FIP4; ARF6*
TGF-beta signaling pathway	0.01218	*TGIF2; SMURF1; SMAD6; FMOD*
Fc gamma R-mediated phagocytosis	0.01354	*PAK1; PAK4; MARCKSL1; GAB2; ARF6*

**Table 6:** Main pathways regulated by miR-1253, miR-1283, miR378e, and miR-579-3p.

Pathways	*p*	Genes
Cellular senescence	3.62 × 10^11^	*NFATC3; PTEN; FOXO3; SIRT1; FOXO1; ZFP36L2; TGFBR1; HIPK3; PPP1CB; PPP2R1B; CDK6; CDK13; CCND1; RBBP4; RAD1*
Signaling pathways regulating pluripotency of stem cells	5.98 × 10^10^	*FZD3; ZFHX3; FZD2; WNT10A; PCGF3; LIF; LIFR; PAX6; IGF1R; REST; KAT6A; IL6ST; SKIL*
Focal adhesion	1.59 × 10^11^	*SHC3; PRKCB; RASGRF1; PTEN; PARVA; IGF2R; PPP1CB; CDC42; PPP1CC; MAPK9; RAP1A; CCND1; PIP5K1A; PAK3; PPP1R12B*

## Data Availability

The datasets generated during this study can be found in the Gene Expression Omnibus (accession number GSE212859).

## Supplementary Materials

The following supporting information can be downloaded at: https://www.mdpi.com/article/10.3390/ijms24065527/s1.

## References

[1] Goldenberg R.L., Culhane J.F., Iams J.D., Romero R. (2008). Epidemiology and causes of preterm birth. Lancet.

[2] Tracy S., Tracy M., Dean J., Laws P., Sullivan E. (2007). Spontaneous preterm birth of liveborn infants in women at low risk in Australia over 10 years: A population-based study. BJOG Int. J. Obstet. Gynaecol..

[3] McPheeters M.L., Miller W.C., Hartmann K.E., Savitz D.A., Kaufman J.S., Garrett J.M., Thorp J.M. (2005). The epidemiology of threatened preterm labor: A prospective cohort study. Am. J. Obstet. Gynecol..

[4] Arpino C., D’Argenzio L., Ticconi C., Di Paolo A., Stellin V., Lopez L., Curatolo P. (2005). Brain damage in preterm infants: Etiological pathways. Ann. Dell’istituto Super. Sanitã.

[5] Beck S. (2010). The world-wide incidence of preterm birth: A systematic review of maternal mortality and morbidity. Bull. World Health Organ..

[6] Mercuro G., Bassareo P.P., Flore G., Fanos V., Dentamaro I., Scicchitano P., Laforgia N., Ciccone M. (2012). Prematurity and low weight at birth as new conditions predisposing to an increased cardiovascular risk. Eur. J. Prev. Cardiol..

[7] Venkatesh K.K., Leviton A., Hecht J.L., Joseph R.M., Douglass L.M., Frazier J.A., Daniels J.L., Fry R.C., O’Shea T.M., Kuban K.C. (2020). Histologic chorioamnionitis and risk of neurodevelopmental impairment at age 10 years among extremely preterm infants born before 28 weeks of gestation. Am. J. Obstet. Gynecol..

[8] Nour N. (2012). Preterm delivery and the millennium development goal. Rev. Obstet. Gynecol..

[9] Lo C.C., Hsu J.J., Hsieh C.C., Hsieh T.T., Hung T.H. (2007). Risk factors for spontaneous preterm delivery before 34 weeks of gestation among Taiwanese women. Taiwan J. Obstet. Gyneco..

[10] Varma R., Gupta J.K., James D.K., Kilby M.D. (2006). Do screening-preventative interventions in asymptomatic pregnancies reduce the risk of preterm delivery--a critical appraisal of the literature. Eur. J. Obstet. Gynecol. Reprod. Biol..

[11] Ancel P.Y. (2004). Perspectives in the prevention of premature birth. Eur. J. Obstet. Gynecol. Reprod. Biol..

[12] Ouyang Y., Mouillet J.-F., Coyne C., Sadovsky Y. (2014). Review: Placenta-specific microRNAs in exosomes—Good things come in nano-packages. Placenta.

[13] http://www.mirbase.org/index.shtml.

[14] Ji L., Brkić J., Liu M., Fu G., Peng C., Wang Y.-L. (2013). Placental trophoblast cell differentiation: Physiological regulation and pathological relevance to preeclampsia. Mol. Asp. Med..

[15] Seitz H., Royo H., Bortolin M.-L., Lin S.-P., Ferguson-Smith A.C., Cavaillé J. (2004). A Large Imprinted microRNA Gene Cluster at the Mouse Dlk1-Gtl2 Domain. Genome Res..

[16] Luo L., Ye G., Nadeem L., Fu G., Yang B.B., Dunk C., Lye S., Peng C. (2012). MicroRNA-378a-5p promotes trophoblast cell survival, migration and invasion by targeting Nodal. J. Cell Sci..

[17] Forbes K., Farrokhnia F., Aplin J., Westwood M. (2012). Dicer-dependent miRNAs provide an endogenous restraint on cytotrophoblast proliferation. Placenta.

[18] Williams Z., Ben-Dov I.Z., Elias R., Mihailovic A., Brown M., Rosenwaks Z., Tuschl T. (2013). Comprehensive profiling of circulating microRNA via small RNA sequencing of cDNA libraries reveals biomarker potential and limitations. Proc. Natl. Acad. Sci. USA.

[19] Renthal N.E., Williams K.C., Mendelson C.R. (2013). MicroRNAs—Mediators of myometrial contractility during pregnancy and labour. Nat. Rev. Endocrinol..

[20] Hromadnikova I., Kotlabova K., Ondrackova M., Pirkova P., Kestlerova A., Novotna V., Hympanova L., Krofta L. (2015). Expression Profile of C19MC microRNAs in Placental Tissue in Pregnancy-Related Complications. DNA Cell Biol..

[21] Elovitz M.A., Brown A.G., Anton L., Gilstrop M., Heiser L., Bastek J. (2014). Distinct cervical microRNA profiles are present in women destined to have a preterm birth. Am. J. Obstet. Gynecol..

[22] Paquette A.G., Shynlova O., Wu X., Kibschull M., Wang K., Price N.D., Lye S.J. (2019). MicroRNA-transcriptome networks in whole blood and monocytes of women undergoing preterm labour. J. Cell Mol. Med..

[23] Winger E.E., Reed J.L., Ji X., Gomez-Lopez N., Pacora P., Romero R. (2020). MicroRNAs isolated from peripheral blood in the first trimester predict spontaneous preterm birth. PLoS ONE.

[24] Sarker S., Scholz-Romero K., Perez A., Illanes S.E., Mitchell M.D., Rice G.E., Salomon C. (2014). Placenta-derived exosomes continuously increase in maternal circulation over the first trimester of pregnancy. J. Transl. Med..

[25] Valadi H., Ekström K., Bossios A., Sjöstrand M., Lee J.J., Lötvall J.O. (2007). Exosome-mediated transfer of mRNAs and microRNAs is a novel mechanism of genetic exchange between cells. Nat. Cell Biol..

[26] Record M. (2014). Intercellular communication by exosomes in placenta: A possible role in cell fusion?. Placenta.

[27] Radnaa E., Richardson L.S., Sheller-Miller S., Baljinnyam T., Silva M.D.C., Kammala A.K., Urrabaz-Garza R., Kechichian T., Kim S., Han A. (2021). Extracellular vesicle mediated feto-maternal HMGB1 signaling induces preterm birth. Lab Chip.

[28] Ghafourian M., Mahdavi R., Jonoush Z.A., Sadeghi M., Ghadiri N., Farzaneh M., Salehi A.M. (2022). The implications of exosomes in pregnancy: Emerging as new diagnostic markers and therapeutics targets. Cell Commun. Signal..

[29] Nair S., Salomon C. (2018). Extracellular vesicles and their immunomodulatory functions in pregnancy. Semin. Immunopathol..

[30] Liu M., Chen Y., Huang B., Mao S., Cai K., Wang L., Yao X. (2018). Tumor-suppressing effects of microRNA-612 in bladder cancer cells by targeting malic enzyme 1 expression. Int. J. Oncol..

[31] Zhu Y., Zhang H.L., Wang Q.Y., Chen M.J., Liu L.B. (2018). Overexpression of microrna-612 restrains the growth, invasion, and tumorigenesis of melanoma cells by targeting espin. PLoS ONE.

[32] Wang M., Wang Z., Zhu X., Guan S., Liu Z. (2019). NFKB1-miR-612-FAIM2 pathway regulates tumorigenesis in neurofibromatosis type 1. Endocrinology.

[33] Garcia-Lacarte M., Martinez J.A., Zulet M.A., Milagro F.I. (2019). Implication of miR-612 and miR-1976 in the regulation of TP53 and CD40 and their relationship in the response to specific weight-loss diets. In Vitro Cell Dev. Biol. Anim..

[34] Gomez-Lopez N., Romero R., Garcia-Flores V., Xu Y., Leng Y., Alhousseini A., Hassan S.S., Panaitescu B. (2017). Amniotic fluid neutrophils can phagocytize bacteria: A mechanism for microbial killing in the amniotic cavity. Am. J. Reprod. Immunol..

[35] https://maayanlab.cloud/Enrichr/enrich?dataset=ca36541ba817bd95fa9ce6f6ead9dd48#.

[36] Wang D., Song W., Na Q. (2012). The emerging roles of placenta-specific microRNAs in regulating trophoblast proliferation during the first trimester. Aust. N. Z. J. Obstet. Gynaecol..

[37] Park S., Lim W., Bazer F.W., Whang K.-Y., Song G. (2018). Quercetin inhibits proliferation of endometriosis regulating cyclin D1 and its target microRNAs in vitro and in vivo. J. Nutr. Biochem..

[38] https://maayanlab.cloud/Enrichr/enrich?dataset=a4c309d2b4db51232173445e28b646b3#.

[39] Polettini J., da Silva M.G. (2020). Telomere-related disorders in fetal membranes associated with birth and adverse pregnancy outcomes. Front. Physiol..

[40] Menon R., Richardson L.S., Lappas M. (2019). Fetal membrane architecture, aging and inflammation in pregnancy and parturition. Placenta.

[41] Li Z., Jia J., Gou J., Zhao X., Yi T. (2014). MicroRNA-451 plays a role in murine embryo implantation through targeting Ankrd46, as implicated by a microarray-based analysis. Fertil. Steril..

[42] Dominguez F., Moreno-Moya J.M., Lozoya T., Romero A., Martínez S., Monterde M., Gurrea M., Ferri B., Núñez M.J., Simón C. (2014). Embryonic miRNA Profiles of Normal and Ectopic Pregnancies. PLoS ONE.

[43] Dixon C.L., Sheller-Miller S., Saade G.R., Fortunato S.J., Lai A., Palma C., Guanzon D., Salomon C., Menon R. (2018). Amniotic Fluid Exosome Proteomic Profile Exhibits Unique Pathways of Term and Preterm Labor. Endocrinology.

[44] Ramos B.R.D., Mendes N.D., Tanikawa A.A., Amador M.A.T., dos Santos N.P.C., dos Santos S.E.B., Castelli E.C., Witkin S.S., da Silva M.G. (2016). Ancestry informative markers and selected single nucleotide polymorphisms in immunoregulatory genes on adverse gestational outcomes: A case control study. BMC Pregnancy Childbirth.

[45] Lilliecreutz C., Larén J., Sydsjö G., Josefsson A. (2016). Effect of maternal stress during pregnancy on the risk for preterm birth. BMC Pregnancy Childbirth.

[46] Menon R., Debnath C., Lai A., Guanzon D., Bhatnagar S., Kshetrapal P.K., Sheller-Miller S., Salomon C., The Garbhini Study Team (2019). Circulating Exosomal miRNA Profile During Term and Preterm Birth Pregnancies: A Longitudinal Study. Endocrinology.

[47] Elovitz M.A., Anton L., Bastek J., Brown A.G. (2015). Can microRNA profiling in maternal blood identify women at risk for preterm birth?. Am. J. Obstet. Gynecol..

[48] Goytain A., Ng T. (2020). NanoString nCounter Technology: High-Throughput RNA Validation. Chimeric RNA Methods Protoc..

[49] Veldman-Jones M.H., Brant R., Rooney C., Geh C., Emery H., Harbron C.G., Wappett M., Sharpe A., Dymond M., Barrett J.C. (2015). Evaluating Robustness and Sensitivity of the NanoString Technologies nCounter Platform to Enable Multiplexed Gene Expression Analysis of Clinical Samples. Cancer Res..

[50] Jacinto S.O.S., Pamplona K., Soares M. (2012). Manual Técnico de Gestação de Alto Risco.

[51] Théry C., Witwer K.W., Aikawa E., Alcaraz M.J., Anderson J.D., Andriantsitohaina R., Antoniou A., Arab T., Archer F., Atkin-Smith G.K. (2018). Minimal information for studies of extracellular vesicles 2018 (MISEV2018): A position statement of the International Society for Extracellular Vesicles and update of the MISEV2014 guidelines. J. Extracell. Vesicles.

[52] Tronco J.A., Ramos B.R.A., Bastos N.M., Alcântara S.A., da Silveira J.C., da Silva M.G. (2020). Alpha-2-macroglobulin from circulating exosome-like vesicles is increased in women with preterm pregnancies. Sci. Rep..

[53] Reis P.P., Tokar T., Goswami R.S., Xuan Y., Sukhai M., Seneda A.L., Móz L.E.S., Perez-Ordonez B., Simpson C., Goldstein D. (2020). A 4-gene signature from histologically normal surgical margins predicts local recurrence in patients with oral carcinoma: Clinical validation. Sci. Rep..

[54] Reis P., Drigo S., Carvalho R., Lapa R.L., Felix T., Patel D., Cheng D., Pintilie M., Liu G., Tsao M.-S. (2020). Circulating mir-16-5p, mir-92a-3p, and mir-451a in plasma from lung cancer patients: Potential application in early detection and a regulatory role in tumorigenesis pathways. Cancers.

[55] Tokar T., Pastrello C., Rossos A.E.M., Abovsky M., Hauschild A.-C., Tsay M., Lu R., Jurisica I. (2018). mirDIP 4.1-integrative database of human microRNA target predictions. Nucleic Acids Res..

[56] Chen E.Y., Tan C.M., Kou Y., Duan Q., Wang Z., Meirelles G.V., Clark N.R., Ma’Ayan A. (2013). Enrichr: Interactive and collaborative HTML5 gene list enrichment analysis tool. BMC Bioinform..

[57] Kuleshov M.V., Jones M.R., Rouillard A.D., Fernandez N.F., Duan Q., Wang Z., Koplev S., Jenkins S.L., Jagodnik K.M., Lachmann A. (2016). Enrichr: A comprehensive gene set enrichment analysis web server 2016 update. Nucleic Acids Res..

[58] Xie Z., Bailey A., Kuleshov M.V., Clarke D.J.B., Evangelista J.E., Jenkins S.L., Lachmann A., Wojciechowicz M.L., Kropiwnicki E., Jagodnik K.M. (2021). Gene set knowledge discovery with Enrichr. Curr. Protoc..

[59] Fan Y., Siklenka K., Arora S.K., Ribeiro P., Kimmins S., Xia J. (2016). miRNet-dissecting miRNA-target interactions and functional associations through network-based visual analysis. Nucleic Acids Res..

[60] Chang L., Zhou G., Soufan O., Xia J. (2020). miRNet 2.0: Network-based visual analytics for miRNA functional analysis and systems biology. Nucleic Acids Res..

[61] Chang L., Xia J. (2023). MicroRNA Regulatory Network Analysis Using miRNet 2.0. Methods Mol. Biol..

